# Mitochondrial event as an ultimate step in ferroptosis

**DOI:** 10.1038/s41420-022-01199-8

**Published:** 2022-10-08

**Authors:** Soo-Jin Oh, Masataka Ikeda, Tomomi Ide, Kyu Yeon Hur, Myung-Shik Lee

**Affiliations:** 1 Department of Health Sciences and Technology, SAIHST, Sungkyunkwan University, Seoul, Korea; 2 Department of Integrated Biomedical Science, Soonchunhyang Institute of Medi-bio Science and Division of Endocrinology, Department of Internal Medicine, Soonchunhyang Medical Center, Soonchunhyang University College of Medicine, Cheonan, Korea; 3 Department of Cardiovascular Medicine, Kyushu University, Fukuoka, Japan; 4 Department of Medicine, Samsung Medical Center, Sungkyunkwan University School of Medicine, Seoul, Korea

**Keywords:** Cell death, Mitochondria

## Abstract

In ferroptosis, the roles of mitochondria have been controversial. To explore the role of mitochondrial events in ferroptosis, we employed mitochondrial DNA-depleted ρ^0^ cells that are resistant to cell death due to enhanced expression of antioxidant enzymes. Expression of mitochondrial-type GPx4 (mGPx4) but no other forms of GPx4 was increased in SK-Hep1 ρ^0^ cells. Likely due to high mGPx4 expression, SK-Hep1 ρ^0^ cells were resistant to ferroptosis by erastin inhibiting xCT channel. In contrast, SK-Hep1 ρ^0^ cells were susceptible to cell death by a high concentration of RSL3 imposing ferroptosis by GPx4 inhibition. Accumulation of cellular ROS and oxidized lipids was observed in erastin- or RSL3-treated SK-Hep1 ρ^+^ cells but not in erastin-treated SK-Hep1 ρ^0^ cells. Mitochondrial ROS and mitochondrial peroxidized lipids accumulated in SK-Hep1 ρ^+^ cells not only by RSL3 but also by erastin acting on xCT on the plasma membrane. Mitochondrial ROS quenching inhibited SK-Hep1 ρ^+^ cell death by erastin or a high dose of RSL3, suggesting a critical role of mitochondrial ROS in ferroptosis. Ferroptosis by erastin or RSL3 was inhibited by a more than 20-fold lower concentration of MitoQ, a mitochondrial ROS quencher, compared to DecylQ, a non-targeting counterpart. Ferroptosis of SK-Hep1 ρ^+^ cells by erastin or RSL3 was markedly inhibited by a VDAC inhibitor, accompanied by significantly reduced accumulation of mitochondria ROS, total peroxidized lipids, and mitochondrial peroxidized lipids, strongly supporting the role of mitochondrial events in ferroptotic death and that of VDAC in mitochondrial steps of ferroptosis induced by erastin or RSL3. SK-Hep1 ρ^+^ cell ferroptosis by sorafenib was also suppressed by mitochondrial ROS quenchers, accompanied by abrogation of sorafenib-induced mitochondrial ROS and mitochondrial peroxidized lipid accumulation. These results suggest that SK-Hep1 ρ^0^ cells are resistant to ferroptosis due to upregulation of mGPx4 expression and mitochondrial events could be the ultimate step in determining final cell fate.

## Introduction

Ferroptosis is characterized by extensive accumulation of peroxidized lipids in iron-dependent manners, which is blocked by iron chelators or lipophilic antioxidants [1]. Ferroptosis is important in the progression of acute kidney injury, acetaminophen-induced liver injury, cardiac ischemia/reperfusion injury, or p53-mediated cell death [2–4].

Several genes including *xCT*, *GPx4*, *ACSL4*, and *LPCAT3* play crucial roles in ferroptosis, and most of them are involved in lipid peroxidation-reduction [5]. Among them, glutathione peroxidase 4 (GPx4) is the only enzyme reducing hydroperoxides in lipoproteins, cholesterol hydroperoxide [6, 7], and phospholipid peroxides [8]. While lipid peroxidation can occur in both cytosol and mitochondria, ferroptosis is reportedly triggered by extra-mitochondrial lipid peroxidation [7]. Furthermore, it has been reported that 100-fold higher concentration of mitochondria-targeting mitoquinone mesylate (MitoQ) was required to prevent ferroptosis compared to non-targeting decylubiquinone (DecylQ) [7], suggesting preferential roles of extra-mitochondrial lipid peroxidation [9].

However, recent investigation demonstrated important roles of mitochondria in ferroptosis [10, 11]. We also have reported a pivotal role of mitochondrial lipid peroxidation and *mGPx4* in doxorubicin-induced ferroptosis of cardiomyocytes [12]. Here, we studied the role of mitochondrial function and *mGPx4* in ferroptosis employing mitochondrial DNA (mtDNA)-depleted SK-Hep1 ρ^0^ cells that show enhanced expression of antioxidant enzymes such as mitochondrial superoxide dismutase, GPx1, or GPx4 [13, 14].

We observed that SK-Hep1 ρ^0^ cells are resistant to ferroptosis due to *mGPx4* induction and obtained evidence suggesting that mitochondrial events are ultimate steps in ferroptosis.

## Results

### Resistance of SK-Hep1 ρ^0^ cells against ferroptosis

**Fig. 1:**
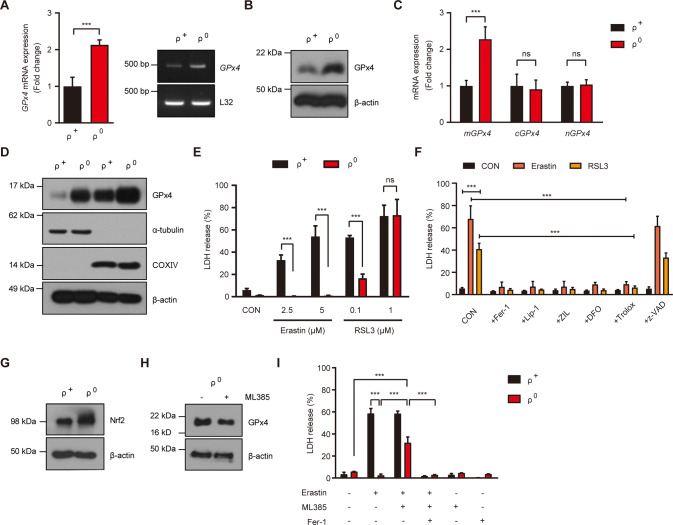
Enhanced GPx4 expression in mtDNA-depleted SK-Hep1 ρ^0^ cells and resistance to cell death by ferroptosis inducers. **A** mRNA expression of *GPx4* in GSK-Hep1 ρ^+^ cells and SK-Hep1 ρ^0^ cells was examined by real-time RT-PCR (left) and RT-PCR (right). **B** GPx4 protein expression in SK-Hep1 ρ^+^ cells and SK-Hep1 ρ^0^ cells were determined by immunoblot analysis using the indicated antibodies. **C** Expression of different isoforms of *GPx4* in SK-Hep1 ρ^+^ cells and ρ^0^ cells were studied by real-time RT-PCR. **D** GPx4 protein levels in the cytoplasmic and mitochondrial fractions were determined using immunoblot analysis. Purity of the cytoplasmic and mitochondrial fractions was confirmed by immunoblot analysis using anti-α-tubulin and -COXIV antibodies, respectively. **E** SK-Hep1 ρ^+^ cells and ρ^0^ cells were treated with 2.5 and 5 µM erastin or 0.1 and 1.0 µM RSL3 for 24 h. Cell death was measured using LDH release assay. **F** SK-Hep1 ρ^+^ cells and ρ^0^ cells were treated with 5 μM erastin or 0.1 μM RSL3 for 24 h in the presence or absence of 2.5 µM Fer-1 (Ferrostatin-1), 0.1 µM Lip-1 (Liproxstatin-1), 10 µM ZIL (Zileuton), 50 µM DFO (Deferoxamine), 50 µM Trolox or 50 µM z-VAD (z-VAD-FMK). Cell death was determined using LDH release assay. **G** Nrf2 protein level in SK-Hep1 ρ^+^ cells and ρ^0^ cells was determined using immunoblot analysis. **H** GPx4 protein level in SK-Hep1 ρ^0^ cell extract was determined using immunoblot analysis with or without treatment with 10 µM ML385, an Nrf2 inhibitor, for 24 h. **I** SK-Hep1 ρ^+^ and ρ^0^ cell death after treatment with 5 µM erastin for 24 h was determined using LDH release assay with or without 10 µM ML385 or 2.5 µM Fer-1 pretreatment for 1 h. Data represent means ± SD from three independent experiments. Data were analyzed by two-tailed Student’s *t* test (**A**, **C**, **E**, ρ^+^ and ρ^0^ cell comparisons in **F**) or one-way ANOVA with Tukey’s multiple comparison test (**F** except ρ^+^ and ρ^0^ cell comparisons, **I**). (CON, control) (*p < 0.05; **p < 0.01; ***p < 0.001; ns, not significant) (Uncropped immunoblots can be found in Fig. S2).

When we studied GPx4 expression, GPx4 mRNA and protein were notably increased in SK-Hep1 ρ^0^ cells compared to parental SK-Hep1 (ρ^+^) cells, consistent with our previous data [14], (Fig. 1A, B). Among GPx4 isoforms, *mitochondrial GPx4* (*mGPx4*) expression was markedly increased in SK-Hep1 ρ^0^ cells compared to SK-Hep1 ρ^+^ cells, while *cytosolic GPx4* (*cGPx4*) or *nuclear GPx4* (*nGPx4*) expression was not different between the two cell types (Fig. 1C). In contrast, fractionation study showed that GPx4 protein expression was enhanced in both cytosolic and mitochondrial fractions, suggesting that enhanced *mGPx4* might lead to increased mGPx4 protein not only in mitochondrial fraction but also in cytoplasmic fraction (Fig. 1D), consistent with our previous data [12].

We next studied whether SK-Hep1 ρ^0^ cells have altered sensitivity to ferroptosis due to enhanced *mGPx4* expression [15]. When we treated SK-Hep1 ρ^0^ cells with erastin, a classical ferroptosis inducer inhibiting xCT [16], SK-Hep1 ρ^0^ cells were remarkably resistant to erastin in a dose range inducing significant SK-Hep1 ρ^+^ cell death (Fig. 1E). We next employed RSL3 inducing ferroptosis through GPx4 inhibition [16]. SK-Hep1 ρ^0^ cells were partially resistant to RSL3 and only a small portion (ca. 15%) of SK-Hep1 ρ^0^ cells underwent cell death at 0.1 μM inducing profound SK-Hep1 ρ^+^ cell death (Fig. 1E). In contrast, SK-Hep1 ρ^0^ cells and ρ^+^ cells were equally sensitive to a higher concentration (1 μM) of RSL3 (Fig. 1E), probably because higher concentrations of RSL3 could overwhelm the increased GPx4 in SK-Hep1 ρ^0^ cells. SK-Hep1 ρ^+^ cell death by erastin or RSL3 was inhibited by authentic ferroptosis inhibitors such as Ferrostatin-1 (Fer-1) or Liproxstatin-1 (Lip-1) [17], a 5-lipoxygenase inhibitor (Zileuton), an iron-chelator (Deferoxamine), and a lipophilic antioxidant (Trolox) but not by z-VAD-FMK (z-VAD) (Fig. 1F), confirming that erastin- or RSL3-induced SK-Hep1 ρ^+^ cell death is caspase-independent ferroptosis.

To study the mechanism of GPx4 induction in SK-Hep1 ρ^0^ cells, we studied Nrf2, a master regulator of antioxidant responses and a transcriptional regulator of GPx4 [18, 19]. Nrf2 expression was markedly increased in SK-Hep1 ρ^0^ cells (Fig. 1G), likely as an adaptive change to mitochondrial stress or reactive oxygen stress (ROS) during ρ^0^ cell derivation [14]. Furthermore, ML385, an Nrf2 inhibitor, not only downregulated GPx4 expression in SK-Hep1 ρ^0^ cells but also significantly attenuated SK-Hep1 ρ^0^ cell resistance to erastin (Fig. 1H, I), supporting the role of Nrf2-dependent *GPx4* induction in SK-Hep1 ρ^0^ cells resistance against ferroptosis [18, 19]. SK-Hep1 ρ^0^ cell death by ML385 was abrogated by Fer-1 (Fig. 1I), indicating that cell death reinstated by ML385 is ferroptosis.

### ROS accumulation in ferroptosis

**Fig. 2:**
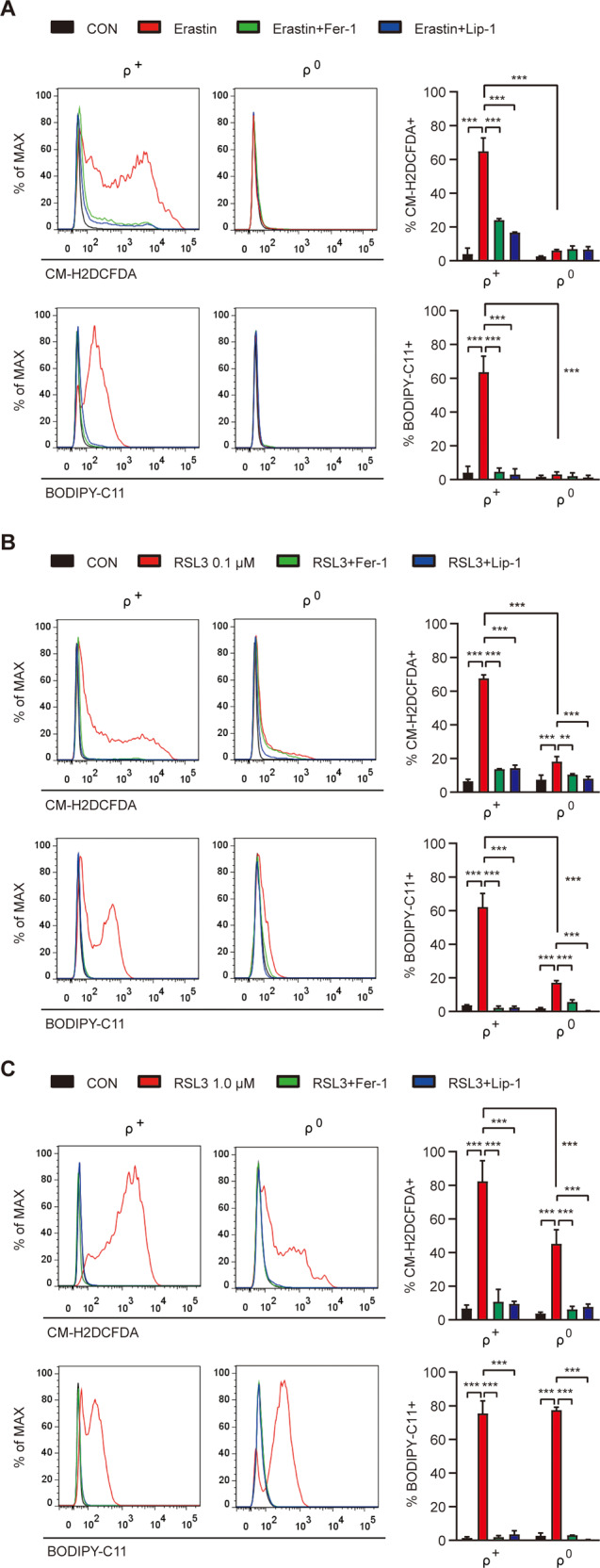
Reduced accumulation of ROS and lipid peroxides in SK-Hep1 ρ^0^ cells. **A** SK-Hep1 ρ^+^ and ρ^0^ cells were treated with 5 μM erastin in the presence or absence of 2.5 μM Fer-1 or 0.1 μM Lip-1 for 16 h and then analyzed for cellular ROS and lipid peroxides by flow cytometry after staining with CM-H2DCFDA and BODIPY-C11, respectively (right). Representative histograms are shown (left panel). **B**, **C** SK-Hep1 ρ^+^ and ρ^0^ cells were treated with 0.1 (**B**) or 1.0 μM (**C**) RSL3 in the presence or absence of 2.5 μM Fer-1 or 0.1 μM Lip-1 for 4 h, and then analyzed for cellular ROS and lipid peroxides, as in **A** (right). Representative histograms are shown (left panel). Data represent means ± SD from three independent experiments. Data were analyzed by one-way ANOVA with Tukey’s multiple comparison test (**A**–**C** except ρ^+^ and ρ^0^ comparison) or two-tailed unpaired Student’s *t* test (ρ^+^ and ρ^0^ cell comparisons in **A**–**C**). (*p < 0.05; **p < 0.01; ***p < 0.001; ****p < 0.0001; ns, not significant).

We next studied intracellular ROS, a critical component of ferroptosis. In erastin-treated SK-Hep1 ρ^+^ cells, massive ROS accumulation was observed by flow cytometry after staining with CM-H2DCFDA, a general ROS sensor (Fig. 2A). In the same cells, massive peroxidized lipid accumulation was also observed by flow cytometry after labeling with BODIPY-C11, a peroxidized lipid sensor (Fig. 2A). ROS or peroxidized lipid accumulation in erastin-treated SK-Hep1 ρ^+^ cells was markedly reduced by Fer-1 or Lip-1 (Fig. 2A), suggesting that ROS or peroxidized lipid accumulation is causally related to erastin-induced ferroptosis. In contrast, accumulation of ROS or peroxidized lipids was absent in erastin-treated SK-Hep1 ρ^0^ cells (Fig. 2A), likely due to high *mGPx4* expression scavenging peroxidized phospholipids.

We next studied ROS accumulation by RSL3 inhibiting GPx4. In 0.1 μM RSL3-treated SK-Hep1 ρ^+^ cells, notable ROS or peroxidized lipid accumulation was seen which was abrogated by Fer-1 or Lip-1 (Fig. 2B), suggesting causal relationship between ROS or peroxidized lipid accumulation and SK-Hep1 ρ^+^ cell ferroptosis. When SK-Hep1 ρ^0^ cells were treated with 0.1 μM RSL3, ROS or peroxidized lipid accumulation was significantly lower (Fig. 2B), consistent with partial resistance of SK-Hep1 ρ^0^ cells against RSL3.

In cells treated with a higher concentration of RSL3 (1 μM), significant ROS or peroxidized lipid accumulation was observed in both SK-Hep1 ρ^+^ cells and ρ^0^ cells (Fig. 2C). In 1 μM RSL3-treated SK-Hep1 ρ^0^ cells, ROS, and peroxidized lipid accumulation was much higher compared to 0.1 μM RSL3 treatment (Fig. 2C), suggesting that *mGPx4* expression in SK-Hep1 ρ^0^ cells could not overcome the effects of 1.0 μM RSL3. Particularly, peroxidized lipid level in 1 μM RSL3-treated SK-Hep1 ρ^0^ cells was similar to that in 1 μM RSL3-treated SK-Hep1 ρ^+^ cells (Fig. 2C), explaining similar SK-Hep1 ρ^0^ cell and ρ^+^ cell death by 1 μM RSL3 (Fig. 1E). ROS or peroxidized lipid accumulation in 1 μM RSL3-treated SK-Hep1 ρ^+^ or ρ^0^ cells was abrogated by Fer-1 or Lip-1 (Fig. 2C).

### Mitochondrial events in ferroptosis

**Fig. 3:**
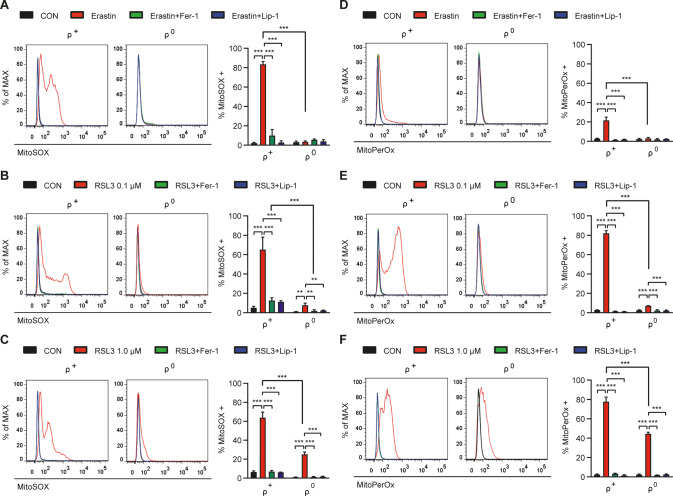
Reduced accumulation of mitochondrial ROS and mitochondrial lipid peroxides in SK-Hep1 ρ^0^ cells. **A** SK-Hep1 ρ^+^ and ρ^0^ cells were treated with 5 μM erastin in the presence or absence of 2.5 μM Fer-1 or 0.1 μM Lip-1 for 16 h and then analyzed for mitochondrial ROS by flow cytometry after staining with MitoSOX (right). Representative histograms are shown (left panel). **B**, **C** SK-Hep1 ρ^+^ and ρ^0^ cells were treated with 0.1 (**B**) or 1.0 μM (**C**) RSL3 in the presence or absence of 2.5 μM Fer-1 or 0.1 μM Lip-1 for 4 h and then analyzed for mitochondrial ROS by flow cytometry after staining with MitoSOX (right). Representative histograms are shown (left panel). **D** SK-Hep1 ρ^+^ cells were treated with 5 μM erastin in the presence or absence of 2.5 μM Fer-1 or 0.1 μM Lip-1 for 16 h and then analyzed for mitochondrial peroxidized lipids by flow cytometry after staining with MitoPerOx (right). Representative histograms are shown (left panel). **E**, **F** SK-Hep1 ρ^+^ and ρ^0^ cells were treated with 0.1 (**E**) or 1.0 μM (**F**) RSL3 in the presence or absence of 2.5 μM Fer-1 or 0.1 μM Lip-1 for 4 h and then analyzed for mitochondrial peroxidized lipids by flow cytometry after staining with MitoPerOx (right). Representative histograms are shown (left panel). Data represent means ± SD from three independent experiments. Data were analyzed by one-way ANOVA with Tukey’s multiple comparison test (**A**–**F** except ρ^+^ and ρ^0^ cell comparisons) or two-tailed unpaired Student’s *t* test (ρ^+^ and ρ^0^ comparisons in **A**–**F**). (*p < 0.05; **p < 0.01; ***p < 0.001; ****p < 0.0001; ns, not significant).

We next studied mitochondrial ROS which are important in several cell death models [20–22], since major site of mGPx4 action in SK-Hep1 ρ^0^ cells might be mitochondria, despite mGPx4 protein expression in both cytoplasm and mitochondria [12]. In erastin-treated SK-Hep1 ρ^+^ cells, significant accumulation of mitochondrial ROS stained with MitoSOX, a mitochondrial ROS probe, was found (Fig. 3A), which could be due to mitochondrial events occurring secondary to the cytosolic events during ferroptosis by erastin primarily affecting xCT on the plasma membrane or possible direct action of erastin on mitochondria such as VDAC opening [23, 24]. Mitochondrial ROS accumulation in erastin-treated SK-Hep1 ρ^+^ cells was abrogated by Fer-1 or Lip-1 (Fig. 3A), supporting that mitochondrial ROS accumulation is crucial in erastin-induced ferroptosis. In erastin-treated SK-Hep1 ρ^0^ cells, mitochondrial ROS accumulation was not observed (Fig. 3A), likely due to mitochondrial ROS scavenging by mGPx4.

In 0.1 μM RSL3-treated SK-Hep1 ρ^+^ cells also, mitochondrial ROS accumulation was well observed which was abrogated by Fer-1 or Lip-1 (Fig. 3B), suggesting a role of mitochondrial ROS in RSL3-induced SK-Hep1 ρ^+^ cell ferroptosis. In 0.1 μM RSL3-treated SK-Hep1 ρ^0^ cells, a little mitochondrial ROS accumulation was observed (Fig. 3B), which was much less than that in 0.1 μM RSL3-treated SK-Hep1 ρ^+^ cells and could explain a minor SK-Hep1 ρ^0^ cell death by 0.1 μM RSL3 (Fig. 1E). Consistently, mitochondrial ROS accumulation in 0.1 μM RSL3-treated SK-Hep1 ρ^0^ cells was abrogated by Fer-1 or Lip-1 (Fig. 3B). In 1.0 μM RSL3-treated SK-Hep1 ρ^0^ cells, significant mitochondrial ROS accumulation was observed, while less than that in 1.0 μM RSL3-treated SK-Hep1 ρ^+^ cells (Fig. 3C), explaining marked SK-Hep1 ρ^0^ cell death by 1.0 μM RSL3. Mitochondrial ROS in 1.0 μM RSL3-treated SK-Hep1 ρ^0^ cells was significantly reduced by Fer-1 or Lip-1 (Fig. 3C), demonstrating mitochondrial ROS accumulation surpassing capacity of induced mGPx4.

We also studied mitochondrial peroxidized lipids which could be effectors in mitochondrial steps of ferroptosis. Staining with MitoPerOx, a BODIPY-C11 derivative detecting mitochondrial inner membrane lipid peroxidation [25], demonstrated a small but significant mitochondrial peroxidized lipid accumulation in erastin-treated SK-Hep1 ρ^+^ cells which was suppressed by Fer-1 or Lip-1 (Fig. 3D), suggesting that mitochondrial lipid peroxidation due to mitochondrial ROS plays crucial roles in ferroptosis. In erastin-treated SK-Hep1 ρ^0^ cells, no accumulation of mitochondrial peroxidized lipids was observed (Fig. 3D), which could be due to mGPx4-mediated inhibition of primary cytosolic event associated with xCT inhibition inducing secondary mitochondrial changes or direct inhibition of erastin-mediated mitochondrial events such as VDAC opening [23]. Mitochondrial peroxidized lipids also accumulated in 0.1 μM RSL3-treated SK-Hep1 ρ^+^ cells which were abrogated by Fer-1 or Lip-1 (Fig. 3E), supporting the role of mitochondrial peroxidized lipids as effectors of RSL3-induced ferroptosis. Mitochondrial peroxidized lipid accumulation in 0.1 μM RSL3-treated SK-Hep1 ρ^0^ cells was significantly lower than that in 0.1 μM RSL3-treated SK-Hep1 ρ^+^ cells (Fig. 3E), consistent with reduced SK-Hep1 ρ^0^ cell death by 0.1 μM RSL3. In 1.0 μM RSL3-treated SK-Hep1 ρ^0^ cells, mitochondrial peroxidized lipids were significantly higher compared to 0.1 μM RSL3 treatment, while still lower than that in 1.0 μM RSL3-treated SK-Hep1 ρ^+^ cells (Fig. 3F). Mitochondrial peroxidized lipids in 1.0 μM RSL3-treated SK-Hep1 ρ^+^ or ρ^0^ cells were abrogated by Fer-1 or Lip-1 (Fig. 3F), as expected.

### Effects of mitochondrial ROS quenching

**Fig. 4:**
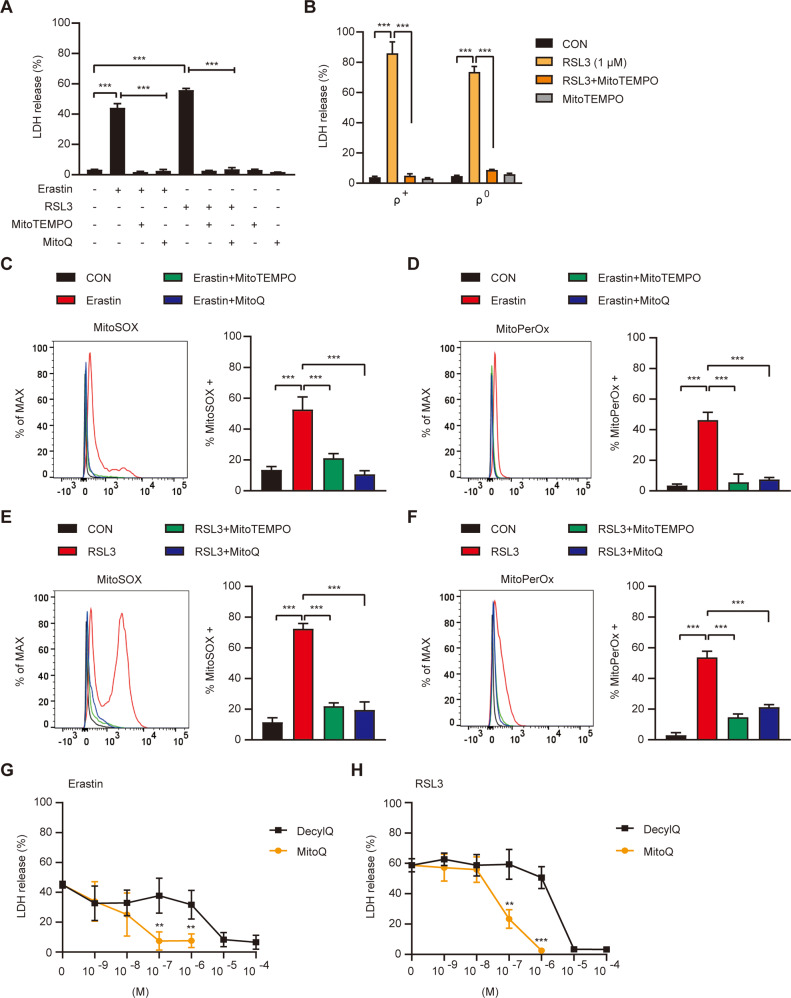
Effect of mitochondrial ROS quenchers on ferroptosis. **A** SK-Hep1 ρ^+^ cells were treated with 5 μM erastin or 0.1 μM RSL3 for 24 h in the presence or absence of 100 μM MitoTEMPO or 500 nM MitoQ. Cell death was determined by LDH release assay. **B** SK-Hep1 ρ^+^ and ρ^0^ cells were treated with 1.0 μM RSL3 in the presence or absence of MitoTEMPO for 24 h. Cell death was determined by LDH release assay. **C**, **D** In SK-Hep1 ρ^+^ treated with 5 μM erastin in the presence or absence of 100 μM MitoTEMPO or 500 nM MitoQ for 16 h, accumulation of mitochondrial ROS (**C**) and mitochondrial lipid peroxides (**D**) was determined by flow cytometry after staining with MitoSOX and MitoPerOx, respectively (right). Representative histograms are shown (left). **E**, **F** In SK-Hep1 ρ^+^ treated with 0.1 μM RSL3 in the presence or absence of 100 μM MitoTEMPO or 500 nM MitoQ for 4 h, accumulation of mitochondrial ROS (**E**) and mitochondrial lipid peroxides (**F**) was determined by flow cytometry after staining with MitoSOX and MitoPerOx, respectively (right). Representative histograms are shown (left). **G**, **H** SK-Hep1 ρ^+^ cells were treated with 5 μM erastin (**G**) or 0.1 μM RSL3 (**H**) for 24 h in the presence or absence of varying concentrations of DecylQ and MitoQ. Cell death was determined by LDH release assay. Data represent means ± SD from three independent experiments. Data were analyzed by one-way ANOVA with Tukey’s multiple comparison test (**A**–**F**) or two-tailed unpaired Student’s *t* test (**G**, **H**). (*p < 0.05; **p < 0.01; ***p < 0.001; ****p < 0.0001; ns, not significant).

We further investigated the role of mitochondrial ROS in ferroptosis by employing mitochondrial ROS scavengers. When SK-Hep1 ρ^+^ cells were pretreated with MitoTEMPO, a mitochondrial ROS quencher, cell death by erastin or 0.1 μM RSL3 was abrogated (Fig. 4A), indicating critical roles of mitochondrial ROS in ferroptosis even in that caused by erastin primarily imposing cytosolic event, while direct effect of erastin on mitochondria cannot be eliminated [24]. SK-Hep1 ρ^+^ cell or ρ^0^ cell death by 1.0 μM RSL3 was also significantly inhibited by MitoTEMPO (Fig. 4B), demonstrating critical roles of mitochondrial ROS or mitochondrial GPx4 inhibition in RSL3-induced SK-Hep1 cell ferroptosis. When we employed MitoQ, a mitochondria-targeted lipophilic ROS quencher [26], SK-Hep1 ρ^+^ cell death by erastin or 0.1 μM RSL3 was again significantly inhibited (Fig. 4A), corroborating the role of mitochondrial ROS in ferroptosis. Consistently, inhibition of erastin- or 0.1 μM RSL3-induced SK-Hep1 ρ^+^ cell death by MitoTEMPO or MitoQ was accompanied by suppression of mitochondrial ROS and mitochondrial lipid oxidation (Fig. 4C–F).

To study the role of mitochondrial ROS more specifically, we compared anti-ferroptotic effects of MitoQ and DecylQ, a non-targeting counterpart [7]. DecylQ inhibited erastin- and RSL3-mediated ferroptosis at more than 100-fold and 20-fold higher concentrations compared to MitoQ, respectively (IC_50_: 8.6 × 10^−7^ vs 5.0 × 10−9 M for erastin and 1.6 × 10^−6^ vs 7.0 × 10^−8^ M for RSL3) (Fig. 4G, H), suggesting more specific and robust effects of mitochondria-targeted antioxidants compared to non-targeted antioxidants and critical roles of mitochondrial events in ferroptosis, in contrast to a previous paper [7]. The difference between inhibition of eratin- or RSL3-induced cell death by DecylQ and that by MitoQ was most conspicuous between 100 nM~1.0 μM (Fig. 4G, H). Furthermore, in this concentration range, MitoQ-mediated reduction of relative accumulation of MitoPerOx by RSL3 was more pronounced compared to that of BODIPY-C11 (Fig. S1A), suggesting that stronger effect of MitoQ on cell death compared to DecylQ is likely due to its preferential effect on mitochondrial peroxidized lipids. However, MitoQ-mediated reduction of erastin-induced accumulation of MitoPerOx was not different from that of BODIPY-C11 (Fig. S1B), suggesting distinct mechanism and kinetics of cytosol-mitochondrial interaction between erastin and RSL3.

**Fig. 5:**
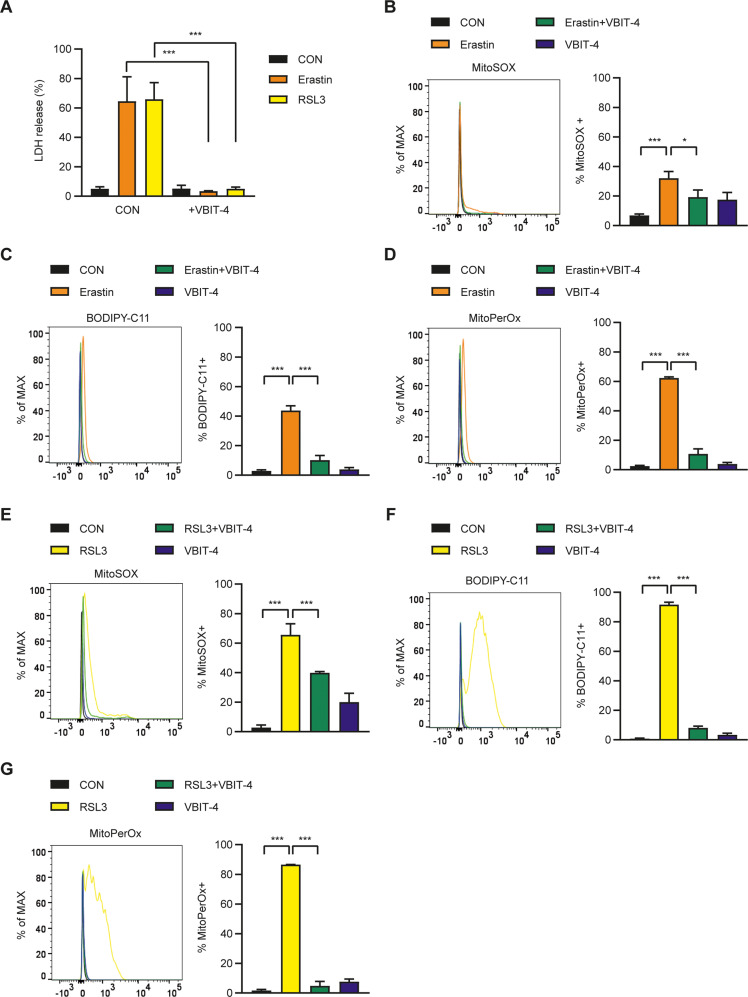
Effect of VDAC inhibitor on ferroptosis. **A** After treatment of SK-Hep1 ρ^+^ cells with 5 μM erastin or 0.1 μM RSL3 in the presence or absence of 10 μM VBIT-4, cell death was evaluated by LDH release assay as in (Fig. 1E). **B**–**D** After treatment of SK-Hep1 ρ^+^ cells with 5 μM erastin in the presence or absence of 10 μM VBIT-4 as in **A**, accumulation of MitoSOX **B**, BODIPY-C11 **C**, and MitoPerOx **D** was evaluated as in (Figs. 3A, 2A, and 3D) (right). Representative histograms are shown (left). **E**–**G** After treatment of SK-Hep1 ρ^+^ cells with 0.1 μM RSL3 in the presence or absence of 10 μM VBIT-4 as in **A**, accumulation of MitoSOX (**E**), BODIPY-C11 (**F**), and MitoPerOx (**G**) was evaluated as in (Figs. 3A, 2A, and 3D) (right). Representative histograms are shown (left). Data represent means ± SD from three independent experiments. Data were analyzed by one-way ANOVA with Tukey’s multiple comparison test (**A**–**G**). (*p < 0.05; **p < 0.01; ***p < 0.001; ****p < 0.0001; ns, not significant).

To further investigate the mechanism of a dominant role of mitochondrial events in ferroptosis, we studied VDAC which can be a link between cytosolic and mitochondrial events [23, 24]. When we employed an antagonist of VDAC, VBIT-4 suppressing VDAC oligomerization and permeabilization [27] almost completely abrogated erastin-induced SK-Hep1 cell death (Fig. 5A), suggesting a critical role of VDAC in erastin-induced ferroptosis. Erastin-induced accumulation of mitochondrial ROS, total peroxidized lipids and mitochondrial peroxidized lipids stained by MitoSOX, BODIPY-C11, and MitoPerOx, respectively, was also markedly suppressed by VBIT-4 (Fig. 5B–D), suggesting roles of VDAC in mitochondrial steps of erastin-induced ferroptosis probably as a linker between cytosolic and mitochondrial events or a direct target of erastin [23, 24]. We also studied the effect of VBIT-4 on RSL3-induced ferroptosis. Here again, RSL3-induced SK-Hep1 cell death was abrogated by VBIT-4 (Fig. 5A). Furthermore, RSL3-induced accumulation of mitochondrial ROS, total peroxidized lipids, and mitochondrial peroxidized lipids stained were markedly suppressed by VBIT-4 (Fig. 5E–G), suggesting roles of VDAC in mitochondrial events in RSL3-mediated ferroptosis as well.

**Fig. 6:**
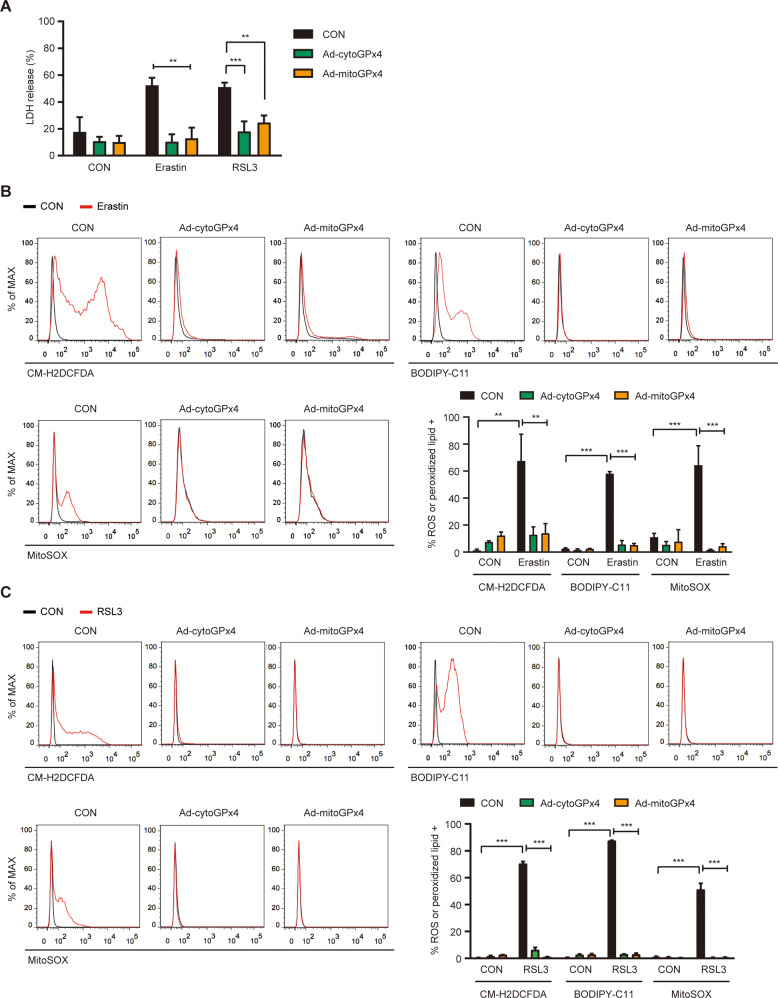
Resistance to cell death and reduced accumulation of cellular ROS, lipid peroxides, or mitochondrial ROS by adenoviral expression of *GPx4*. **A** After infection of SK-Hep1 ρ^+^ cells with adenovirus expressing Ad-cytoGPx4 or Ad-mitoGPx4, cells were treated with 5 μM erastin or 0.1 μM RSL3 for 24 h. Cell death was measured by LDH release assay. **B**, **C** After treatment of SK-Hep1 ρ^+^ cells infected with adenovirus expressing Ad-cytoGPx4 or Ad-mitoGPx4) with 5 μM erastin (**B**) or 0.1 μM RSL3 (**C**), accumulation of cellular ROS, peroxidized lipids and mitochondrial ROS was determined by flow cytometry after staining with CM-H2DCFDA, BODIPY-C11 and MitoSOX, respectively (right lower). Representative histograms are shown (upper and left lower panels). Data represent means ± SD from three independent experiments. Data were analyzed by one-way ANOVA with Tukey’s multiple comparison test. (*p < 0.05; **p < 0.01; ***p < 0.001; ****p < 0.0001; ns, not significant).

To prove the dominant role of mitochondrial events in ferroptosis, we infected SK-Hep1 ρ^+^ cells with adenovirus expressing *cGPx4* (Ad-cytoGPx4-FLAG) or *mGPx4* (Ad-mitoGPx4-FLAG) [12]. Adenoviral *mGPx4* expression almost completely abrogated SK-Hep1 ρ^+^ cell death by erastin or 0.1 μM RSL3 (Fig. 6A), suggesting critical roles of mGPx4 and mitochondrial events of erastin- or RSL3-induced ferroptosis. Accumulation of ROS, peroxidized lipids, or mitochondrial ROS by erastin or RSL3 was also abrogated by adenoviral *mGPx4* expression (Fig. 6B, C). When we infected cells with Ad-cytoGPx4-FLAG expressing *cGPx4*, SK-Hep1 ρ^+^ cell death and accumulation of ROS, peroxidized lipids or mitochondrial ROS by erastin or RSL3 were also significantly inhibited (Fig. 6A–C), which could be due to inhibition of cytosolic events of ferroptosis inducing dominant mitochondrial event, although significant roles of cytosolic event itself in ferroptosis cannot be disregarded.

### Resistance of SK-Hep1 ρ^0^ cells against sorafenib

**Fig. 7:**
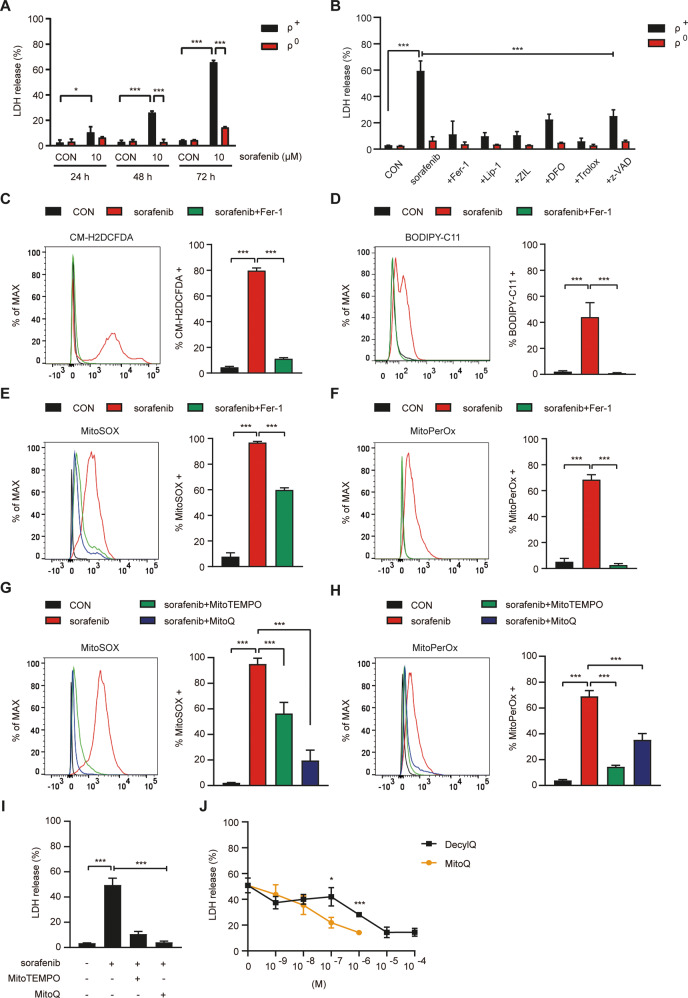
Resistance of SK-Hep1 ρ^0^ cells against sorafenib-induced ferroptosis. **A** SK-Hep1 ρ^+^ and ρ^0^ cells were treated with 10 μM sorafenib for the indicated periods, and cell death was determined using LDH release assay. **B** SK-Hep1 ρ^+^ and ρ^0^ cells were treated with 10 μM sorafenib in the presence or absence of 2.5 µM Fer-1, 0.1 µM Lip-1, 10 µM ZIL, 50 µM DFO, 50 µM Trolox or 50 µM z-VAD for 72 h. Cell death was determined using an LDH release assay. **C**, **D** In SK-Hep1 ρ^+^ and ρ^0^ cells treated with 10 μM sorafenib in the presence or absence of Fer-1 for 48 h, accumulation of cellular ROS (**C**) and lipid peroxides (**D**) was analyzed by flow cytometry after staining with CM-H2DCFDA and BODIPY-C11, respectively (right). Representative histograms are shown (left). **E**, **F** In SK-Hep1 ρ^+^ and ρ^0^ cells treated with 10 μM sorafenib in the presence or absence of 2.5 μM Fer-1 for 48 h, accumulation of mitochondrial ROS (**E**) and mitochondrial lipid peroxides (**F**) was determined by flow cytometry after staining with MitoSOX and MitoPerOx, respectively (right). Representative histograms are shown (left). **G**, **H** In SK-Hep1 ρ^+^ and ρ^0^ cells treated with 10 μM sorafenib in the presence or absence of 100 μM MitoTEMPO or 500 nM MitoQ for 48 h, accumulation of mitochondrial ROS (**G**) and mitochondrial lipid peroxides (**H**) was determined by flow cytometry after staining with MitoSOX and MitoPerOx, respectively (right). Representative histograms are shown (left). **I** After treatment of SK-Hep1 ρ^+^ cells with 10 μM sorafenib in the presence or absence of 100 μM MitoTEMPO or 500 nM MitoQ, cell death was evaluated using LDH release assay. **J** After treatment of SK-Hep1 ρ^+^ cells with 10 μM sorafenib in the presence or absence of varying doses of DecylQ or MitoQ, cell death was evaluated using LDH release assay. Data represent means ± SD from three independent experiments. Data were analyzed by one-way ANOVA with Tukey’s multiple comparison test (**A**–**I** except ρ^+^ and ρ^0^ cell comparisons) or two-tailed unpaired Student’s *t* test (ρ^+^ and ρ^0^ cell comparisons in **A**, **J**). (*p < 0.05; **p < 0.01; ***p < 0.001; ****p < 0.0001; ns, not significant).

We finally studied the potential clinical significance of ρ^0^ cell resistance to ferroptosis by employing sorafenib, an anti-cancer drug inducing ferroptosis [28]. Sorafenib-induced marked SK-Hep1 ρ^+^ cell death (Fig. 7A), which was significantly inhibited by Fer-1, Lip-1, Zileuton, Deferoxamine, Trolox, or z-VAD (Fig. 7B), suggesting that sorafenib induces SK-Hep1 cell death through both ferroptosis and apoptosis. In contrast, SK-Hep1 ρ^0^ cells were resistant to sorafenib-induced cell death (Fig. 7A). Sorafenib-induced ROS or peroxidized lipid accumulation in SK-Hep1 ρ^+^ cells which was abrogated by Fer-1 (Fig. 7C, D), supporting sorafenib-induced ferroptosis.

We also studied whether mitochondrial events are important in ferroptosis by sorafenib inhibiting xCT [29, 30], similar to erastin. Again similar to erastin, marked mitochondrial ROS and mitochondrial peroxidized lipid accumulation were observed in sorafenib-treated SK-Hep1 cells (Fig. 7E, F). Mitochondrial ROS or mitochondrial peroxidized lipid accumulation in sorafenib-treated SK-Hep1 cells was significantly reduced by Fer-1 (Fig. 7E, F), supporting roles of mitochondrial events in sorafenib-induced ferroptosis. Furthermore, MitoTEMPO or MitoQ significantly inhibited mitochondrial ROS or mitochondrial peroxidized lipid accumulation by sorafenib (Fig. 7G, H), which was accompanied by suppression of sorafenib-induced SK-Hep1 cell death (Fig. 7I), again suggesting roles of mitochondrial events in sorafenib-induced ferroptosis. Furthermore, DecylQ inhibited sorafenib-mediated cell death at more than 10-fold higher concentration compared to MitoQ (IC_50_: 1.5 × 10^−7^ vs 1.3 × 10^−8^ M) (Fig. 7J), which indicates more specific and robust effects of mitochondria-targeted antioxidant compared to non-targeted antioxidant in sorafenib-induced ferroptosis caused by inhibition of xCT on the plasma membrane and suggests ultimate roles of mitochondrial events in ferroptosis.

## Discussion

mtDNA-depleted ρ^0^ cells [31] are valuable tools to study mitochondrial functions, ROS, cell death or inflammation [14, 32, 33]. Since GPx4 is a critical component in ferroptosis and GPx4 expression was increased in SK-Hep1 ρ^0^ cells [14], we investigated whether SK-Hep1 ρ^0^ cells were resistant to ferroptosis and also studied the role of mitochondria in ferroptosis.

We observed that SK-Hep1 ρ^0^ cells were resistant to cell death by erastin or a low concentration of RSL3, accompanied by a markedly reduced ROS or peroxidized lipids, which is different from previous data showing no difference in ferroptosis between parental and ρ^0^ cells [34, 35]. These discrepancies could be ascribed to differences in cell types, methods of ρ^0^ cell generation, or the level of antioxidant gene expression in ρ^0^ cells.

SK-Hep1 ρ^0^ cell resistance to ferroptosis together with no ROS or peroxidized lipid accumulation by erastin primarily inducing cytosolic event of ferroptosis could be due to mGPx4 scavenging peroxidized lipids not only in mitochondria but also in the cytoplasm [12]. *mGPx4* induction could increase cytosolic GPx4 during transportation into mitochondria [12]. In addition to GPx4, a high expression of GPx1 detoxifying peroxidized lipids other than phospholipids in SK-Hep1 ρ^0^ cells [36, 37] might contribute to the reduction of ROS and peroxidized lipids.

Besides cytoplasmic action of mGPx4, mGPx4 might act on mitochondrial events as well because mitochondrial ROS or mitochondrial peroxidized lipid accumulated in erastin-treated SK-Hep1 ρ^+^ cells and mitochondrial ROS quenchers inhibited erastin-induced SK-Hep1 ρ^+^ cell death together with abrogated mitochondrial ROS or mitochondrial peroxidized lipid accumulation. Mitochondrial events by erastin could be due to direct action on mitochondria such as VDAC opening and changed mitochondrial outer membrane permeability [23, 24], or secondary to the cytoplasmic event of ferroptosis inducing mitochondrial change such as BID transactivation [38].

In contrast to erastin inhibiting xCT on the plasma membrane, RSL3 induces ferroptosis through GPx4 inhibition and intracellular peroxidized phospholipid generation [15]. Mitochondrial ROS and mitochondrial peroxidized lipid accumulation in RSL3-treated SK-Hep1 ρ^+^ cells could be due to the occurrence of cytosolic events of ferroptosis associated with RSL3-mediated cytosolic GPx4 inhibition leading to secondary mitochondrial changes through cellular events such as BID transactivation [38], similar to the erastin treatment. Instead, direct inhibition of mitochondrial GPx4 by RSL3 might lead to similar consequences of mitochondrial ROS and mitochondrial peroxidized lipid accumulation. Protection of RSL3-induced cell death by mitochondrial ROS quenchers accompanied by abrogation of mitochondrial ROS or mitochondrial peroxidized lipid accumulation indicates that mitochondrial events are ultimate steps in ferroptotic death, whether mitochondrial ROS was produced by secondary mitochondrial changes following RSL3-mediated cytosolic GPx4 inhibition or by direct mitochondrial event of RSL3-mitochondrial GPx4 inhibition.

We observed that VBIT-4, a VDAC antagonist, significantly inhibited SK-Hep1 cell death by erastin or RSL3, accompanied by markedly reduced accumulation of mitochondrial ROS, total peroxidized lipids, and mitochondrial peroxidized lipids, strongly supporting a critical role of mitochondrial events in ferroptosis. These data are in line with previous reports showing erastin effect on VDAC [23, 24] or blockade of glutamate-induced oxytosis or acetaminophen-induced hepatic ferroptosis by a VDAC inhibitor [39, 40]. However, these data are dissimilar to previous papers showing no role of VDAC3 in RSL3-induced ferroptosis [41]. In contrast, degradation of VDAC2/3 and carbonylation of VDAC2 by RSL3 have been reported [42, 43], suggesting the potential role of VDAC in RSL3-mediated ferroptosis depending on cell types or context. VDAC might play a role in the transport of iron from the cytoplasm to mitochondria [44], and thereby contribute to mitochondrial steps of ferroptosis regardless of inducers.

Inhibition of erastin-induced ferroptosis by adenoviral *mGpx4* expression also suggests that mitochondrial ROS could be critical, similar to the inhibition of erastin-induced ferroptotic cell death by MitoTEMPO or MitoQ. Previous papers have reported exclusive mGPx4 localization in the mitochondrial fraction [45]. However, since we observed that GPx4 from adenoviral *mGPx4* expression was found in the cytoplasm during transportation of mitochondrial GPx4 [12], our adenoviral *mGPx4* expression experiment alone does not confirm the sole role of mitochondrial event in ferroptosis. Nonetheless, another data showing the significant ferroptosis inhibition by mitochondrial ROS quenchers alone supports that mitochondrial events are critical steps in ferroptosis.

**Fig. 8:**
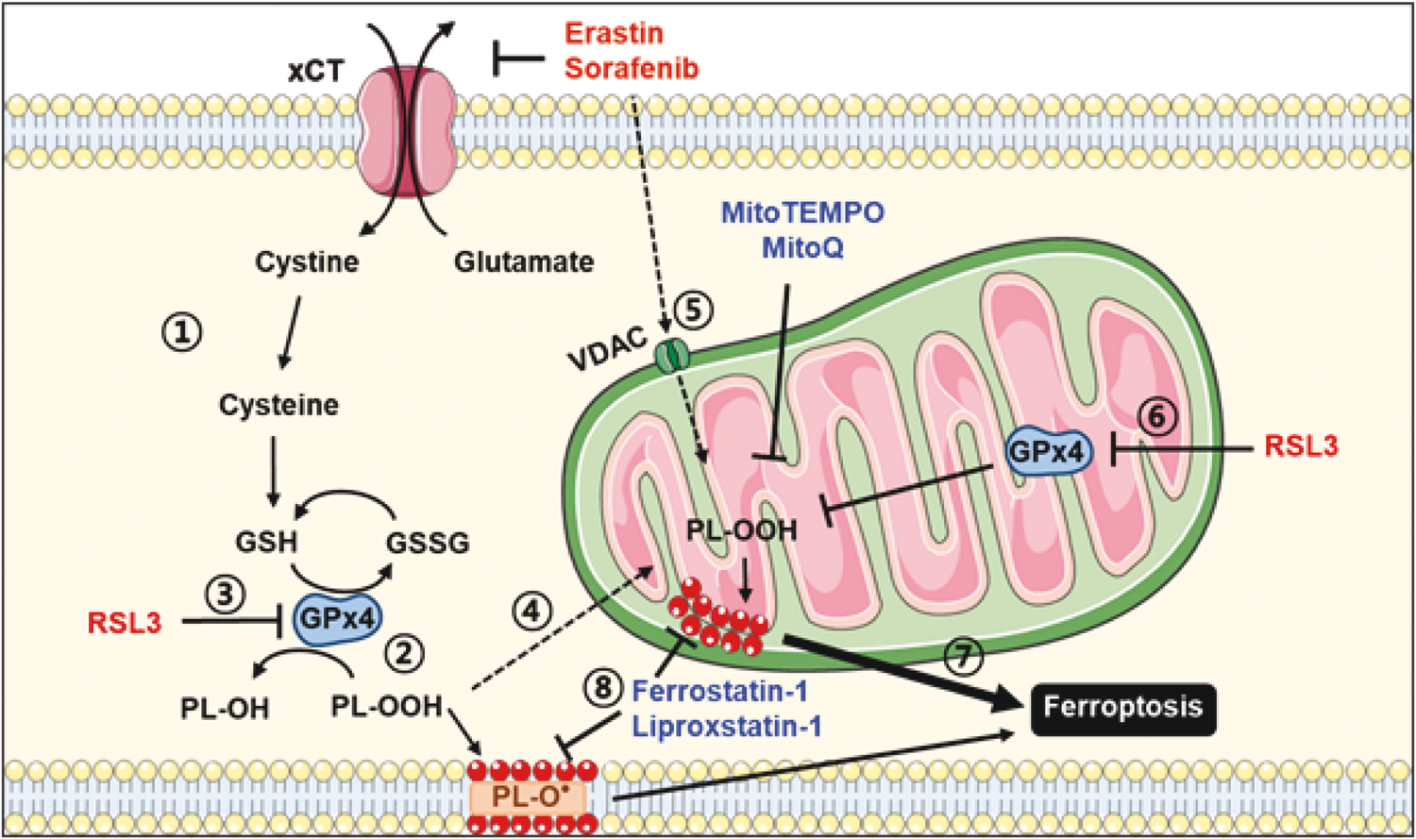
Schematic diagram showing the role of cytosolic or mitochondrial events and accumulation of ROS or oxidized lipids in ferroptosis. Inhibition of xCT by erastin or sorafenib leads to depletion of cytoplasmic cysteine and GSH (①), a cofactor for GPx4 detoxifying peroxidized phospholipids (PL-OOH) (②). RSL3 directly inhibits cytosolic GPx4 (③). Mitochondrial events of ferroptosis may follow the cytosolic events of ferroptosis through diverse pathways of cytosol-mitochondrial interaction such as BID transactivation (④). Inducers of cytosolic events of ferroptosis may directly act on mitochondrial targets such as VDAC, evoking mitochondrial events of ferroptosis (⑤). GPx4 inhibitors may also inhibit mitochondrial GPx4 (⑥). Mitochondrial events of ferroptosis are likely a dominant player in the execution of ferroptotic death (⑦) because inhibition of mitochondrial events alone with mitochondria-specific antioxidants can abrogate ferroptotic death even that by ferroptosis inducers primarily inhibiting xCT on the plasma membrane, and mitochondria-targeting lipophilic antioxidants have much higher potency against ferroptosis compared to non-targeting counterparts. Ferrostatin-1 and liproxstatin-1 are radical-trapping antioxidants (RTAs) potently inhibiting ferroptosis (⑧). Art figure was made partly by recombining the images of the Creative Commons (creativecommons.org) released for free.

Furthermore, more efficient inhibition of erastin- or RSL3-induced ferroptosis by MitoQ compared to DecylQ suggests that mitochondrial events could be dominant players in ferroptosis, whether mitochondrial ROS was produced by secondary mitochondrial changes following cytosolic events of ferroptosis (e.g., xCT inhibition followed by BID activation or cytosolic GPx4 inhibition) or by direct mitochondrial events (e.g., mitochondrial GPx4 inhibition or VDAC opening) (Fig. 8). Our data suggesting that mitochondrial ROS and mitochondrial lipid peroxidation are the key events in ferroptosis is in contrast to previous papers reporting that extra-mitochondrial lipid peroxidation is more important than mitochondrial lipid peroxidation in ferroptosis [7] or no accumulation of mitochondrial ROS by erastin [34]. Our results are also different from previous data showing a more efficient blockade of ferroptosis by DecylQ compared to MitoQ, suggesting a minor role of mitochondrial peroxidation in MEF ferroptosis [7]. Such differences could be due to discrepancies in the treatment method, types of ROS inducers, or species of phospholipids studied. Different cells might have distinct susceptibility to mitochondrial ROS or mitochondrial lipid peroxidation depending on cellular characteristics including expression of DHODH modulating mitochondrial ubiquinol [11].

We observed SK-Hep1 ρ^0^ cell resistance to sorafenib which is widely used as a chemotherapeutic agent against hepatoma [28]. Although papers refuting ferroptosis in sorafenib-induced cancer cell death have been published [46], our data showing inhibition of sorafenib-induce SK-Hep1 cell death by Fer-1 or Lip-1 together with significantly reduced lipid peroxidation strongly supports sorafenib-induced ferroptosis. Furthermore, suppression of sorafenib-induced cell death by MitoTEMPO or MitoQ accompanied by reduced mitochondrial ROS or mitochondrial peroxidized lipids suggests that mitochondrial events play crucial roles in ferroptosis induced by xCT inhibitors such as erastin or sorafenib [29, 30, 47].

Altogether, our data suggest that mitochondrial events such as mitochondrial ROS or mitochondrial peroxidized lipid accumulation following cytosolic events by ferroptosis inducers through cytosol-mitochondria interaction or those due to direct mitochondrial effects of ferroptosis inducers play ultimate roles in ferroptotic death (Fig. 8), which could be important in cancer cell death by anti-cancer chemotherapy. Avoidance of such mitochondrial events could be a mechanism of cancer cells with aberrant or deficient mitochondrial function to resist ferroptosis by chemotherapeutic agents.

## Materials and methods

### Cell culture

SK-Hep1 cells obtained from American Type Culture Collection (ATCC) were grown in DMEM medium (LM-001-05, Welgene, Korea)−1% penicillin-streptomycin-amphotericin B mixture (17-745E, Lonza, Basel, Swiss) supplemented with 10% fetal bovine serum (Corning, Corning, NY, USA). SK-Hep1 ρ^0^ cells were derived by culturing SK-Hep1 cells in the presence of 100 ng/ml ethidium bromide for >20 generations [13, 37], and then maintained in the presence of 50 μg/ml uridine. Cells were tested for mycoplasma contamination using a Mycoplasma PCR Detection Kit (*e*-Myco^TM^, 25236, iNtRON Biotechnology, Korea).

### Cell death assay

Cell death was assessed using LDH release assay (G1780, Promega^TM^ Corporation, Madison, WI, USA). Briefly, cells seeded 1 × 10^4^ well in a 96-well plate were exposed to various concentrations of cytotoxic compounds and inhibitors for 24 h. The culture supernatant was collected and analyzed according to the manufacturer’s protocol.

### Antibodies and reagent

Erastin (S7242) and RSL3 (S8155), Lip-1 (S7699), VBIT-4 (S3544), Mitoquinone mesylate (MitoQ, S8978), Decylubiquione (DecylQ, D7911) were purchased from Selleck Chemicals (Houston, TX, USA). Fer-1 (SML0583), Deferoxamine (DFO, D9533), Trolox (238813), z-VAD-FMK (V116), ML385 (SML1833), MitoTEMPO (SML0737) were purchased from Sigma-Aldrich (St. Louis, MO, USA). Zileuton (3308) was from Tocris Bioscience (Bristol, UK). The following antibodies were used: GPx4 (ab125066, Abcam, Cambridge, UK) and Nrf2 (ab62352, Abcam), COXIV (4844, Cell signaling Technology, Danvers, MA, USA), FLAG (F3165, Sigma-Aldrich), α-tubulin (sc-8035, Santa Cruz, Dallas, TX, USA) and β-actin (sc-47778, Santa Cruz).

### RT-PCR and real-time RT-PCR

RNA was isolated using TRIzol reagent (Invitrogen, Waltham, MA, USA). cDNA synthesis was performed using 1 μg of total RNA and M-MLV Reverse Transcriptase (M1701, Promega). RT-PCR was performed using PCR PreMix (K-2016, BIONEER, Korea)and specific primers [14] for 35 cycles. Quantitative RT-PCR was performed using SYBR Green Master Mix (RR420A, Takara Bio, Kusatsu, Shiga, Japan) and specific primers in a QuantStudio 3 Real-Time PCR machine (Applied Biosystems, Waltham, MA, USA). Gene expression was normalized to that of RPL32 mRNA using the 2^−ΔCt^ method. *cGPx4* mRNA level was determined by subtracting the 2^−ΔCt^ values of *mGPx4* and *nGPx4* from that of total *GPx4*.

Primers sequences were as follows:

GPx4 forward (for RT-PCR), TGT GCG CGC TCC ATG CAC GAG T;

GPx4 reverse (for RT-PCR), AAA TAG TGG GGC AGG TCC TTC TCT;

Total GPx4 forward (for quantitative RT-PCR), GTT TTC CGC CAA GGA CAT CG;

Total GPx4 reverse (for quantitative RT-PCR), ACT TCG GTC TTG CCT CAC TG;

mitochondrial GPx4 forward, ATT GGT CGG CTG GAC GAG;

mitochondrial GPx4 reverse, TCG ATG TCC TTG GCG GAA AA;

nuclear GPx4 forward, CAG CGG TGC CAG AGC C;

nuclear GPx4 forward, GGA CTT GGG ACA TTC GTG GA;

RPL32 forward, CAT CCG GCA CCA GTC AGA CC;

RPL32 reverse, TGT GAG CGA TCT CGG CAC AG.

### Measurement of ROS

Intracellular ROS and peroxidized lipid levels were determined using CM-H2DCFDA (C6827, Life Technologies, Grand Island, NY, USA) and BODIPY^TM^ 581/591 C11 (D3861, Life Technologies), respectively. Mitochondrial ROS and mitochondrial lipid peroxide levels were determined using MitoSOX^TM^ Red (M36008, Life Technologies) and MitoPerOx (18798, Cayman Chemical, Ann Arbor, MI, USA), respectively. After incubation of cells with dye at 37 °C for 30 min, flow cytometry was conducted on BD FACSVerse and FACSCanto II (BD Biosciences, San Jose, CA, USA). Data analysis was performed using FlowJo software (7.6.5, FlowJo, LLC, BD Biosciences).

### Adenoviral infection

Ad-cytoGPx4-FLAG and Ad-mitoGPx4-FLAG recombinant adenoviral vectors were transfected into HEK293AD cells using Lipofectamine 2000 (11668027, Thermo Fisher Scientific, Waltham, MA, USA) for 7~10 days. Cells were then collected and subjected to three cycles of freezing at −80 °C and thawing at 37 °C. After vortexing, recombinant adenovirus containing cytoGPx4 or mitoGPx4 genes was harvested from the supernatant by centrifugation. SK-Hep1 cells were infected with Ad-cytoGPx4-FLAG or Ad-mitoGPx4-FLAG adenovirus at an MOI of 20 for 24 h for further experiments.

### Cell fractionation

Cells were fractionated into mitochondria and cytoplasm using a mitochondrial/cytoplasmic fractionation kit (ab65320, Abcam), according to the manufacturer’s instructions. Briefly, cells were homogenized by a pestle homogenizer, and then were centrifuged at 700 × *g* for 10 min. The supernatant was re-centrifuged at 10,000 × *g* for 30 min to collect supernatant (cytoplasmic fraction) and pellet (mitochondrion fraction). The purity of cytosolic and mitochondrial fractions was confirmed by immunoblot analysis using anti-α-tubulin and -COXIV antibodies, respectively.

### Immunoblot analysis

Cell extracts lysed in RIPA buffer [50 mM Tris/HCl, pH 7.4, 150 mM NaCl, 1% NP-40, 0.5% sodium deoxycholate, 0.1% SDS supplemented with 1% PMSF and cOmplete^TM^ protease inhibitor cocktail (11697498001, Roche, Basel, Swiss)] was centrifuged at 13,500 rpm, 4 °C for 15 min. Protein concentrations were determined by the Bradford method employing a kit (Bio-Rad Laboratories Inc., Hercules, CA, USA). Protein samples were separated on an SDS-PAGE gel (6–12%) and transferred onto PVDF membranes (Millipore, Burlington, MA, USA), which were incubated with primary antibodies in 5% nonfat skim milk-Tris-0.05% Tween 20 at 4 °C overnight. Immunoreactive bands were visualized by sequential incubation with HRP (horseradish peroxidase)-labeled secondary antibody (Cell Signaling Technology) and EZ-Western Lumi F ECL solution (DoGenBio Co., Korea).

### Statistical analysis

Data were analyzed using the two-tailed Student’s *t* test or one-way analysis of variance followed by Tukey’s multiple comparison test. Data are presented as means ± SD of three independent experiments. *p* values <0.05 were considered significant.

## Supplementary information

Figure S1

Figure S2

## Data Availability

The data generated or analyzed during the current study and materials are available from the corresponding author without imposed restriction on reasonable request.

## References

[1] Shimada K, Skouta R, Kaplan A, Yang WS, Hayano M, Dixon SJ (2016). Global survey of cell death mechanisms reveals metabolic regulation of ferroptosis. Nat Chem Biol.

[2] Fearnhead HO, Vandenabeele P, Vanden Berghe T (2017). How do we fit ferroptosis in the family of regulated cell death?. Cell Death Differ.

[3] Jiang L, Kon N, Li T, Wang SJ, Su T, Hibshoosh H (2015). Ferroptosis as a p53-mediated activity during tumour suppression. Nature.

[4] Xie Y, Hou W, Song X, Yu Y, Huang J, Sun X (2016). Ferroptosis: process and function. Cell Death Differ.

[5] Forcina GC, Dixon SJ (2019). GPX4 at the crossroads of lipid homeostasis and ferroptosis. Proteomics.

[6] Brigelius-Flohé R, Maiorino M (2013). Glutathione peroxidases. Biochim Biophys Acta.

[7] Friedmann Angeli JP, Schneider M, Proneth B, Tyurina YY, Tyurin VA, Hammond VJ (2014). Inactivation of the ferroptosis regulator Gpx4 triggers acute renal failure in mice. Nat Cell Biol.

[8] Cozza G, Rossetto M, Bosello-Travain V, Maiorino M, Roveri A, Toppo S (2018). Glutathione peroxidase 4-catalyzed reduction of lipid hydroperoxides in membranes: the polar head of membrane phospholipids binds the enzyme and addresses the fatty acid hydroperoxide group toward the redox center. Free Radic Biol Med.

[9] Kagan VE, Mao G, Qu F, Angeli JP, Doll S, Croix CS (2017). Oxidized arachidonic and adrenic PEs navigate cells to ferroptosis. Nat Chem Biol.

[10] Gao M, Yi J, Zhu J, Minikes AM, Monian P, Thompson CB (2019). Role of mitochondria in ferroptosis. Mol Cell.

[11] Mao C, Liu X, Zhang Y, Lei G, Yan Y, Lee H (2021). DHODH-mediated ferroptosis defence is a targetable vulnerability in cancer. Nature.

[12] Tadokoro T, Ikeda M, Ide T, Deguchi H, Ikeda S, Okabe K (2020). Mitochondria-dependent ferroptosis plays a pivotal role in doxorubicin cardiotoxicity. JCI Insignt.

[13] Kim JY, Kim YH, Chang I, Kim S, Pak YK, Oh BH (2002). Resistance of mitochondrial DNA-deficient cells to TRAIL: role of Bax in TRAIL-induced apoptosis. Oncogene.

[14] Park SY, Chang I, Kim J-Y, Kang S-W, Park S-H, Singh K (2004). Resistance of mitochondrial DNA-depleted cells against cell death: Role of mitochondrial superoxide dismutase. J Biol Chem.

[15] Yang WS, SriRamaratnam R, Welsch ME, Shimada K, Skouta R, Viswanathan VS (2014). Regulation of ferroptotic cancer cell death by GPX4. Cell.

[16] Yang WS, Stockwell BR (2016). Ferroptosis: death by lipid peroxidation. Trends Cell Biol.

[17] Zilka O, Shah R, Li B, Angeli JF, Griesser M, Conrad M (2017). On the mechanism of cytoprotection by ferrostatin‑1 and liproxstatin‑1 and the role of lipid peroxidation in ferroptotic cell death. ASC Cent Sci.

[18] Chen G-H, Song C-C, Pantopoulos K, Wei X-L, Zheng H, Luo Z (2022). Mitochondrial oxidative stress mediated Fe-induced ferroptosis via the NRF2-ARE pathway. Free Radic Biol Med.

[19] Singh A, Venkannagari S, Oh KH, Zhang Y-Q, Rohde JM, Liu L (2016). Small molecule inhibitor of NRF2 selectively intervenes therapeutic resistance in KEAP1-deficient NSCLC tumors. ACS Chem Biol.

[20] Kagan VE, Tyurin VA, Jiang J, Tyurina YY, Ritov VB, Amoscato AA (2005). Cytochrome c acts as a cardiolipin oxygenase required for release of proapoptotic factors. Nat Chem Biol.

[21] Nakagawa T, Shimizu S, Watanabe T, Yamaguchi O, Otsu K, Yamagata H (2005). Cyclophilin D-dependent mitochondrial permeability transition regulates some necrotic but not apoptotic cell death. Nature.

[22] Wang H, Liu C, Zhao Y, Gao G (2020). Mitochondria regulation in ferroptosis. Eur J Cell Biol.

[23] DeHart DN, Fanga D, Heslopa K, Li L, Lemaster JJ, Maldonado EN (2018). Opening of voltage dependent anion channels promotes reactive oxygen species generation, mitochondrial dysfunction and cell death in cancer cells. Biochem Pharm.

[24] Yagoda N, von Rechenberg M, Zaganjor E, Bauer AJ, Yang WS, Fridman DJ (2007). RAS-RAF-MEK-dependent oxidative cell death involving voltage-dependent anion channels. Nature.

[25] Prime TA, Forkink M, Logan A, Finichiu PG, McLachlan J, Pun PBL (2012). A ratiometric fluorescent probe for assessing mitochondrial phospholipid peroxidation within living cells. Free Radic Biol Med.

[26] Dashdorj A, Jyothi KR, Lim S, Jo A, Nguyen MN, Ha J (2013). Mitochondria-targeted antioxidant MitoQ ameliorates experimental mouse colitis by suppressing NLRP3 inflammasome-mediated inflammatory cytokines. BMC Med.

[27] Kim J, Gupta R, Blanco LP, Yang S, Steinfer-Kuzmine A, Wang K (2019). VDAC oligomers form mitochondrial pores to release mtDNA fragments and promote lupus-like disease. Science.

[28] Chen S, Zhu J-Y, Zang X, Zhai Y-Z (2021). The emerging role of ferroptosis in liver diseases. Front Cell Dev Biol.

[29] Chen D, Fan Z, Rauh M, Buchfelder M, Eyupoglu IY, Savaskan N (2017). ATF4 promotes angiogenesis and neuronal cell death and confers ferroptosis in a xCT-dependent manner. Oncogene.

[30] Li Y, Yan H, Xu X, Liu H, Wu C, Zhao L (2020). Erastin/sorafenib induces cisplatin-resistant non-small cell lung cancer cell ferroptosis through inhibition of the Nrf2/xCT pathway. Oncol Lett.

[31] King MP, Attardi G (1989). Human cells lacking mtDNA: repopulation with exogenous mitochondria by complementation. Science.

[32] Ishikawa K, Takenaga K, Akimoto M, Koshikawa N, Yamaguchi A, Imanishi H (2008). ROS-generating mitochondrial DNA mutations can regulate tumor cell metastasis. Science.

[33] Nakahira K, Haspel JA, Rathinam VAK, Lee S-J, Dolinay T, Lam HC (2011). Autophagy proteins regulates innate immune responses by inhibiting the release of mitochondrial DNA mediated by the NALP3 inflammasome. Nat Immunol.

[34] Dixon SJ, Lemberg KM, Lamprecht MR, Skouta R, Zaitsev EM, Gleason CE (2012). Ferroptosis: an iron-dependent form of nonapoptotic cell death. Cell.

[35] Gaschler MM, Hu F, Feng H, Linkermann A, Min W, Stockwell BR (2018). Determination of the subcellular localization and mechanism of action of ferrostatins in suppressing ferroptosis. ASC Chem Biol.

[36] de Haan JB, Witting PK, Stefanovic N, Pete J, Daskalakis M, Kola I (2006). Lack of the antioxidant glutathione peroxidase-1 does not increase atherosclerosis in C57BL/J6 mice fed a high-fat diet. J Lipid Res.

[37] Park SY, Choi GH, Choi HI, Ryu J, Jung CY, Lee W (2005). Depletion of mitochondrial DNA causes impaired glucose utilization and insulin resistance in L6 GLUT4myc myocytes. J Biol Chem.

[38] Neitemeier S, Jelinek A, Laino V, Hoffmann L, Eisenbach I, Eying IR (2017). BID links ferroptosis to mitochondrial cell death pathways. Redox Biol.

[39] Nagakannan P, Islam MI, Karimi-Abdolrezaee S, Eftekharpour E (2019). Inhibition of VDAC1 protects against glutamate-induced oxytosis and mitochondrial fragmentation in hippocampal HT22 cells. Cell Mol Neurobiol.

[40] Niu B, Lei X, Xu Q, Ju Y, Xu D, Mao L (2022). Protecting mitochondria via inhibiting VDAC1 oligomerization alleviates ferroptosis in acetaminophen-induced acute liver injury. Cell Biol Toxicol.

[41] Yang WS, Stockwell BR (2008). Synthetic lethal screening identifies compounds activating iron-dependent, nonapoptotic cell death in oncogenic-RAS-harboring cancer cells. Chem Biol.

[42] Chen Y, Liu Y, Lan T, Qin W, Zhu Y, Qin K (2018). Quantitative profiling of protein carbonylations in ferroptosis by an aniline-derived probe. J Am Chem Soc.

[43] Yang Y, Luo M, Zhang K, Zhang J, Gao T, O’ Connell D (2020). Nedd4 ubiquitylates VDAC2/3 to suppress erastin-induced ferroptosis in melanoma. Nat Commun.

[44] Lipper CH, Stofleth JT, Bai F, Sohn YS, Roy S, Mittler R (2019). Redox-dependent gating of VDAC by mitoNEET. Proc Natl Acad Sci USA.

[45] Arai M, Imai H, Koumura T, Yoshida M, Emoto K, Umeda M (1999). Mitochondrial phospholipid hydroperoxide glutathione peroxidase plays a major role in preventing oxidative injury to cells. J Biol Chem.

[46] Zheng J, Sato M, Mishima E, Sato H, Proneth B, Conrad M (2021). Sorafenib fails to trigger ferroptosis across a wide range of cancer cell lines. Cell Death Dis.

[47] Dixon SJ, Patel DN, Welsch M, Skouta R, Lee ED, Hayano M (2014). Pharmacological inhibition of cystine-glutamate exchange induces endoplasmic reticulum stress and ferroptosis. eLife.

