# Correction: Mitochondrial event as an ultimate step in ferroptosis

**DOI:** 10.1038/s41420-022-01223-x

**Published:** 2022-10-20

**Authors:** Soo-Jin Oh, Masataka Ikeda, Tomomi Ide, Kyu Yeon Hur, Myung-Shik Lee

**Affiliations:** 1 Department of Health Sciences and Technology, SAIHST, Sungkyunkwan University, Seoul, Korea; 2 Department of Integrated Biomedical Science, Soonchunhyang Institute of Medi-bio Science and Division of Endocrinology, Department of Internal Medicine, Soonchunhyang Medical Center, Soonchunhyang University College of Medicine, Cheonan, Korea; 3 Department of Cardiovascular Medicine, Kyushu University, Fukuoka, Japan; 4 Department of Medicine, Samsung Medical Center, Sungkyunkwan University School of Medicine, Seoul, Korea

**Keywords:** Cell death, Mitochondria

Correction to: *Cell Death Discovery*, 10.1038/s41420-022-01199-8, published online 08 October 2022

The original version of this article contained an error. In line 11 of the Introduction, “cholesterol phospholipids” should be “cholesterol hydroperoxide”. The authors apologize for the error. The original article has been corrected.

