# Supplementary material for: Mitochondrial event as an ultimate step in ferroptosis
Source: Cell Death Discov. 2022 Oct 8;8:414. doi: 10.1038/s41420-022-01199-8 (PMC9547870; doi:10.1038/s41420-022-01199-8)

**Fig. S1. Effect of concentration of MitoQ on accumulation of BODIPY-C11 and MitoPerOx. A,B** After treatment of SK-Hep1 cells with 0.1 μM RSL3 (**A**) or 5 μM erastin (**B**) in the presence or absence of 10^-6^ ~ 10^-9^ M MitoQ as in (**Fig 4G-H**), accumulation of BODIPY-C11 and MitoPerOx was evaluated as in (**Fig. 5D-G**). Data represent means ± SD from three independent experiments. Data were analyzed by one-way ANOVA with Tukey’s multiple comparison test. (**p* < 0.05; ***p* < 0.01; ****p* < 0.001; *****p* < 0.0001; ns, not significant)


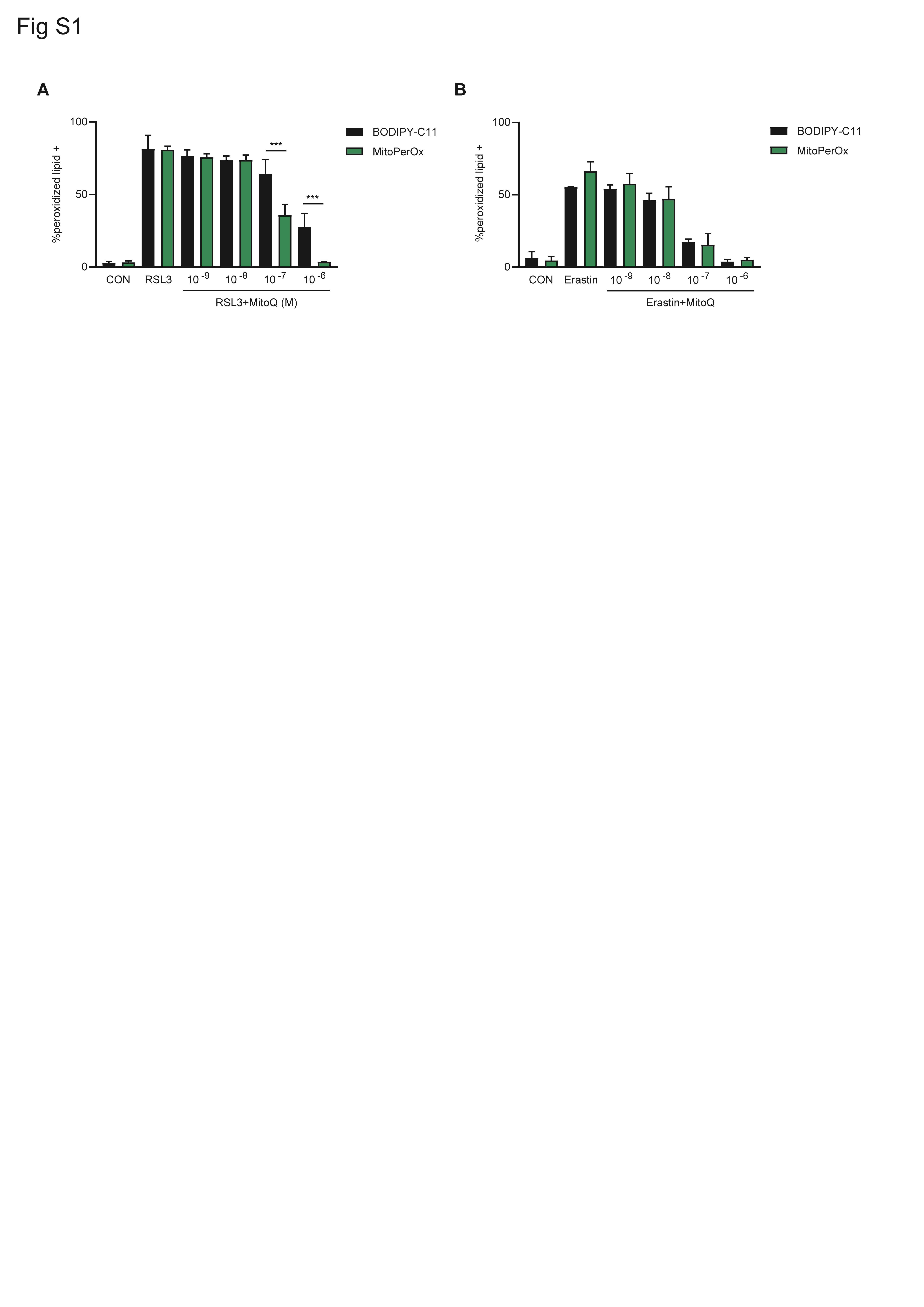

Supplement: Supplementary file 1 — Figure S1 [file 41420_2022_1199_MOESM1_ESM.docx]
