# Supplementary figures and images for: Mitochondrial event as an ultimate step in ferroptosis
Source: Cell Death Discov. 2022 Oct 8;8:414. doi: 10.1038/s41420-022-01199-8 (PMC9547870; doi:10.1038/s41420-022-01199-8)

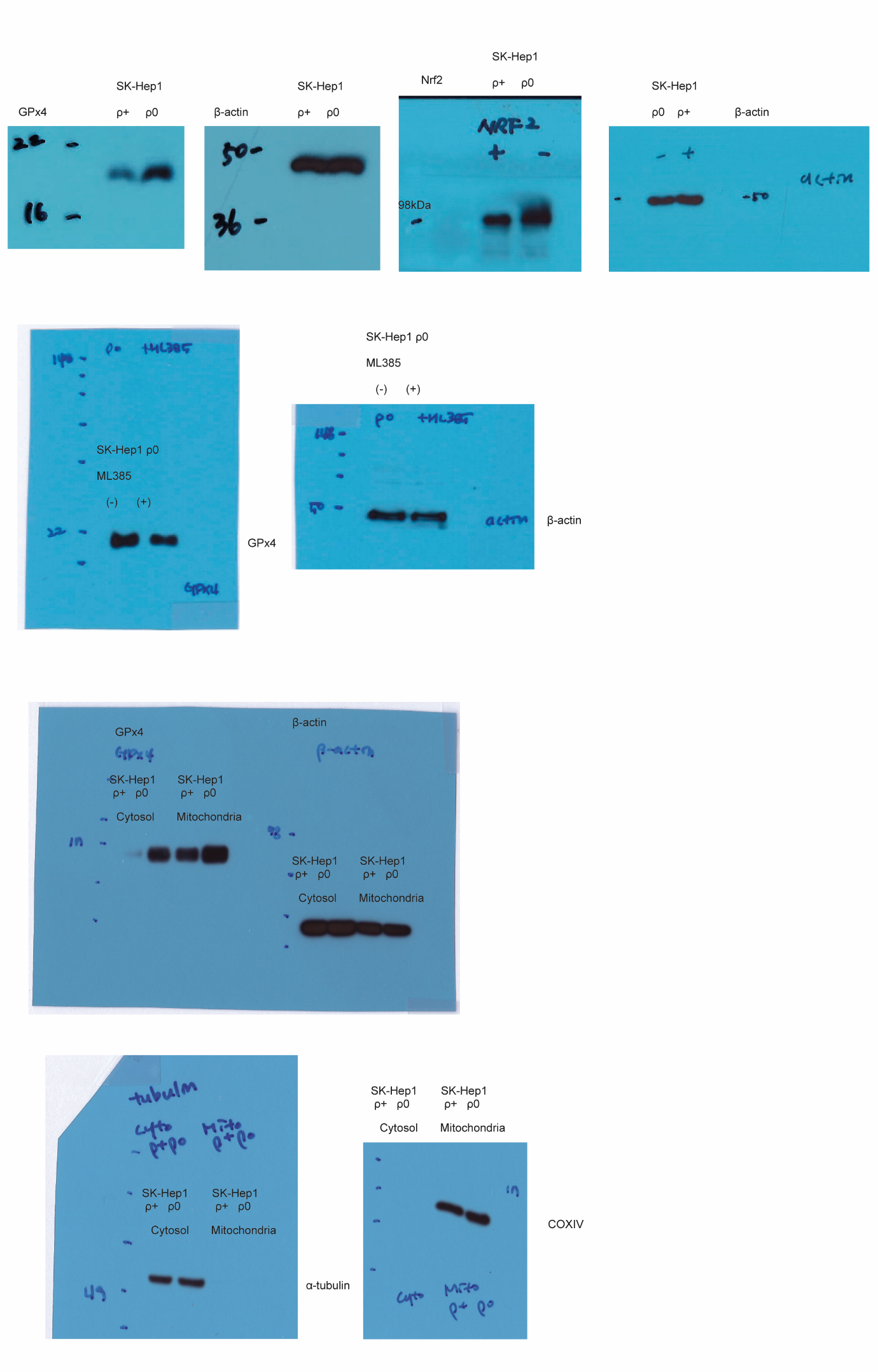


**Fig. S2. Uncropped gel of immunoblots.**

Supplement: Supplementary file 2 — Figure S2 [file 41420_2022_1199_MOESM2_ESM.docx]
